# The effect of increased standardized ileal digestible lysine through increased soybean meal during late gestation on sow lactation performance

**DOI:** 10.1093/tas/txaf108

**Published:** 2025-08-02

**Authors:** Abigail K Jenkins, Jason C Woodworth, Jordan T Gebhardt, Robert D Goodband, Mike D Tokach, Joel M DeRouchey

**Affiliations:** Department of Animal Sciences and Industry, College of Agriculture, Kansas State University, Manhattan, KS 66506-0201, USA; Department of Animal Sciences and Industry, College of Agriculture, Kansas State University, Manhattan, KS 66506-0201, USA; Department of Diagnostic Medicine/Pathobiology, College of Veterinary Medicine, Kansas State University, Manhattan, KS 66506-0201, USA; Department of Animal Sciences and Industry, College of Agriculture, Kansas State University, Manhattan, KS 66506-0201, USA; Department of Animal Sciences and Industry, College of Agriculture, Kansas State University, Manhattan, KS 66506-0201, USA; Department of Animal Sciences and Industry, College of Agriculture, Kansas State University, Manhattan, KS 66506-0201, USA

**Keywords:** gestating sow, lactating sow, lysine, sow milk yield, soybean meal

## Abstract

A total of 87 sows (Line 241, DNA) and their offspring were used to evaluate the effects of increasing standardized ileal digestible (SID) Lys in late gestation diets on lactating sow and litter performance. Sows were blocked by parity and body weight (BW) on day 90 of gestation and allotted to one of three treatments with 29 replications per treatment. Diets included increasing dietary SID Lys (0.60, 0.80, or 1.00%) accomplished by increasing soybean meal (14, 21, or 29% of the diet). Sows were allowed 2.04 kg/d of their treatment diet from day 90 of gestation until farrowing for average SID Lys intakes of 11.9, 15.8, or 19.9 g/d. After farrowing, sows had ad libitum access to a common lactation diet containing 1.10% SID Lys. Urine samples were collected on day 90 and 110 of gestation to determine urinary creatinine levels. Litters were cross-fostered within dietary treatment by 48 h after farrowing to equalize litter size. Parity group was included in the statistical model as a fixed effect with classifications of primiparous (n = 35) or multiparous (n = 52) sows. Weight gain from day 90 to 110 of gestation increased (linear, *P* < 0.001) as SID Lys increased. Change in urinary creatinine level from day 90 to 110 of gestation tended to decrease (linear, *P* = 0.063) as SID Lys increased suggesting that muscle catabolism decreased with increasing SID Lys. There were no differences in starting litter size or piglet birth weight with increasing SID Lys in late gestation. Piglet average daily gain (ADG) from day 2 to 10 of lactation increased (linear, *P *= 0.017) as SID Lys increased. From day 2 until weaning, litters from sows fed 15.8 g/d of SID Lys in gestation had the greatest (quadratic, *P* = 0.044) litter weight gain. Pre-weaning mortality from birth until day 2 of lactation was greatest for sows fed 15.8 g/d of SID Lys (quadratic, *P* = 0.025). There was a parity group × gestation diet interaction (*P* = 0.049) for pre-weaning mortality from day 2 to weaning where mortality increased as SID Lys increased in primiparous sows but decreased in multiparous sows. However, the differences in mortality did not influence the number of pigs weaned per treatment. In conclusion, increased SID Lys through increased soybean meal linearly increased late gestation sow BW gain and piglet ADG during early lactation. Litters from sows fed 15.8 g/d of SID Lys had the greatest litter ADG during late lactation and overall.

## Teaser

Sows fed 15.8 g/d of standardized ileal digestible lysine from day 90 of gestation until farrowing had greater litter weight gain during lactation compared with sows fed 11.9 or 19.9 g/d of standardized ileal digestible lysine.

## INTRODUCTION

Total pigs born per litter has steadily increased as genetic selection continues to produce more prolific sows. During late gestation, there is an increase in both fetal and mammary growth with Lys being the first limiting amino acid (AA) for both. Due to the rise in litter size it has been hypothesized that today’s prolific sow may have an increased requirement for Lys in late gestation (Feyera and Theil, 2017). For this reason, interest in defining the Lys requirements of late gestating sows has intensified. The NRC (2012) estimates the requirement for standardized ileal digestible (SID) Lys intake to be 16.7 g/d after day 90 of gestation for primiparous sows, and to decrease as parity increases, with a requirement estimate of 11.5 g/d for fourth parity or older sows. Various indicators have been used to estimate the SID Lys requirement of late gestating sows including whole body protein deposition, AA oxidation, and total piglets born alive with SID Lys requirement estimates ranging from 11.0 to 18.7 g/d depending on parity and the response criteria used (Samuel et al., 2012; Thomas et al., 2021a; Jo and Kim, 2023).

Because of the substantial increase in fetal and mammary growth during late gestation, Farmer et al. (2022) investigated the effects of increased SID Lys in late gestation and observed that a 40% increase above NRC (2012) requirement estimates stimulated mammary development in primiparous sows. Increasing SID Lys through increased soybean meal (SBM) intake in late gestation led to enhanced fetal and mammary growth at day 110 of gestation, as indicated by increased parenchymal mass which led to greater parenchymal protein, DNA, and RNA (Farmer et al., 2022). However, when a second study was conducted utilizing the same treatment structure with multiparous sows, there was no improvement in fetal or mammary growth as SID Lys increased (Farmer et al., 2023). These two studies ended on day 110 of gestation and the effects of the increased SID Lys in late gestation on lactation performance were not investigated. Recently, Johannsen et al. (2024; multiparous sows) and Kloostra et al. (2025; primiparous sows) have investigated the effects of increasing SID Lys intake through additional SBM on lactation performance. Both observed increases in milk production when providing approximately 21 to 23 g/d SID Lys intake.

Therefore, the objective of this trial was to determine if increased SID Lys through increased SBM fed from day 90 of gestation until farrowing improves lactating sow and litter performance. We hypothesized that increased SID Lys through increased SBM would result in improved milk production due to changes in mammary gland development as evidenced improved litter growth during lactation.

## MATERIALS AND METHODS

### Animals and Treatment Structure

The protocol used in this experiment was approved by the Kansas State University Institutional Animal Care and Use Committee (IACUC # 4485.46). This study was conducted at the Kansas State University Swine Teaching and Research Center in Manhattan, KS. During gestation, sows were housed in group pens by farrowing group with pens containing approximately 30 sows. Gestation pens had completely slatted concrete floors and allowed approximately 2.14 m^2^/sow. Gestation pens were equipped with two nipple waterers and an electronic feeding system (Gestal 3G Feeding System, Jyga Technologies, St-Lambert-de-Lauzon, Quebec, Canada) to allow measure of individual feed intakes. During lactation, sows were housed individually in an environmentally controlled and mechanically ventilated barn. Farrowing stalls were equipped with an electronic feeding system (Gestal Quattro Feeders, Jyga Technologies, St-Lambert-de-Lauzon, Quebec, Canada) and an individual cup waterer. Farrowing stalls also contained a heated mat for piglet comfort.

A total of 87 sows (Line 241, DNA Genetics, Columbus, NE) were used across three batch farrowing groups from May to September 2023. On approximately day 90 of gestation, sows were weighed, then blocked by body weight (BW) and parity and assigned to one of three dietary treatments. Two corn-soybean meal-based diets were formulated to contain 0.60 or 1.00% SID Lys via two levels of SBM (14 or 29% of the diet) and feed grade L-Lys (0.10 or 0.15% of diet; Table 1). The electronic feeding system was used to blend the two diets (50:50 blend) to provide the intermediate 0.80% SID Lys diet. Sows were allowed approximately 2.04 kg/d of their treatment diet from day 90 of gestation until farrowing for average SID Lys intakes of 11.9, 15.8, or 19.9 g/d. Sows were moved to the farrowing house on day 110 of gestation but remained on their treatment diet until the day they farrowed at which time they were switched to ad libitum access to a common lactation diet containing 1.10% SID Lys. All diets for this trial were manufactured at the Kansas State University O.H. Kruse Feed Technology Innovation Center (Manhattan, KS). All diets were formulated to meet or exceed NRC (2012) nutrient requirement estimates (Table 1) and were fed in meal form. Samples of gestation diets were collected upon movement of each group of sows from gestation to the farrowing barn (day 110 of gestation). Lactation diets were collected on the day before weaning each group of sows. The electronic feeding systems in gestation pens and farrowing stalls were calibrated weekly for each diet in order to ensure accuracy of feed allocation and measurement. Nitrogen content of complete feed samples was determined by combustion using a Leco TruMac N with TruMac operating software (Leco Corporation, St. Joseph, MI). A protein factor of 6.25 was used to convert N content to crude protein (Table 1).

**Table 1.** Diet composition (as-fed basis)

	Gestation^1^		
Item	Low Lys	High Lys	Lactation
Ingredient, %			
Corn	82.30	67.55	65.95
Soybean meal, 47.7% CP	14.00	28.75	28.15
Soybean oil	---	---	2.00
Calcium carbonate	1.15	1.10	1.25
Monocalcium P, 21.5% P	1.35	1.15	1.15
Sodium chloride	0.50	0.50	0.50
L-Lys-HCl	0.10	0.15	0.30
DL-Met	0.05	0.15	0.08
L-Thr	0.05	0.16	0.11
L-Trp	---	---	0.01
Trace mineral premix	0.25	0.25	0.25
Vitamin premix	0.25	0.25	0.25
Total	100.00	100.00	100.00
Calculated analysis			
Standardized ileal digestible (SID) amino acids			
Lys, %	0.60	1.00	1.10
Ile:Lys	76	70	63
Leu:Lys	189	148	132
Met:Lys	42	42	31
Met and Cys:Lys	76	69	55
Thr:Lys	75	75	63
Trp:Lys	20.6	20.5	19
Val:Lys	88	77	68
His:Lys	54	47	41
Total Lys, %	0.70	1.14	1.23
ME, kcal/kg	3,265	3,261	3,358
NE, kcal/kg^2^	2,549	2,522	2,615
SID Lys:NE, g/Mcal	2.36	3.96	4.21
CP, %	13.6	19.6	19.3
Ca, %	0.80	0.79	0.89
P, %	0.60	0.63	0.62
STTD P, %	0.50	0.50	0.50
Chemical analysis			
CP, %	11.4	18.4	18.3

^1^Diets were fed from day 90 of gestation until farrowing. Low and high Lys diets were blended at a 50:50 ratio to form the intermediate treatment.

^2^NE value for soybean meal (91.4% of corn) was derived based on Sotak-Peper et al. (2015), who used the equation from Noblet et al. (1994) to determine NE from ME. Other ingredient NE values were based on NRC (2012).

Sow BW was measured on day 90 and 110 of gestation, after farrowing, and at weaning. Sow backfat depth and loin depth were measured on day 90 and 110 of gestation and at weaning at the last rib, 8 cm from the midline using a real-time ultrasound machine (IBEX Pro, E.I. Medical Imaging, Loveland, CO). Sow caliper score (Knauer and Baitinger, 2015) was measured at the last rib on day 90 and 110 of gestation and at weaning. Pigs were cross fostered by 48 h of birth within sow treatment to equalize litter size. Pigs were individually weighed within 24 h after birth (day 1), on day 2 and 10 of lactation, and at weaning. Weaning occurred at an average age of 17.8 days (range: 16 to 20 days). Creep feed and supplemental milk were not offered to litters throughout this study.

Coefficient of variation (CV) of pig weight within a litter was calculated on day 2, day 10, and at weaning by dividing the standard deviation of the average pig weight of the litter by the average pig weight of the litter. Milk yield was estimated using the model developed by Hansen et al. (2012). This model used litter size at weaning and litter average daily gain (ADG) to estimate daily milk yield from day 2 until weaning. Total milk yield was calculated as the summation of daily milk yield from day 2 of lactation until weaning. Total milk yield was then divided by the difference of weaning age from day 2 to determine average milk yield per day.

### Colostrum and Urine Analysis

Colostrum was collected from each sow from the 3^rd^, 4^th^, and 5^th^ teats on either side of the underline within 1 h of the start of farrowing to determine crude protein content. The birth of the first piglet in the litter was considered as start of farrowing. Nitrogen content of colostrum samples was determined by combustion using a Leco TruMac N with TruMac operating software (Leco Corporation, St. Joseph, MI). The N content was multiplied by 6.38 to determine crude protein content (AOAC, 2023). Sow urine samples were collected on day 90 and 110 of gestation to determine urinary creatinine levels. Sow urine samples were collected via the method described by Nickel et al. (2017). Urine was extracted into a conical tube containing 1 mL of HCl to prevent volatilization of N. All samples were frozen and stored at -20 °C until analysis. Urinary creatinine content was measured via a urinary creatinine colorimetric assay kit (Cayman Chemical Company, Ann Arbor, MI).

### Statistical Analyses

Performance data for the trial were analyzed using the lme4 package of R (Version 4.0.0, R Foundation for Statistical Computing, Vienna, Austria) as a randomized complete block design. Blocking structure accounted for parity and BW. In sows fed 11.9 g/d of SID Lys, there were 12, 7, 5, and 5 sows in parity category 1, 2, 3, and 4, respectively. In both the 15.8 and 19.9 g/d SID Lys treatments, each treatment included 11, 8, 5, and 5 parity 1, 2, 3, and 4 parity sows, respectively. Sow served as the experimental unit. Treatment and parity group (multiparous or primiparous sows) and their interaction were included as fixed effects while farrowing group was included as a random intercept. Count data were analyzed using a Poisson distribution using a logit link function. Proportion data, including litter born alive, stillborn, born mummified, and pre-weaning mortality were analyzed with a binomial distribution using a logit link function. Change in urinary creatinine from day 90 to 110 of gestation was analyzed using the lme4 package of R with treatment, parity group, and their interaction included as fixed effects with plate and group included as a random effect. Preplanned linear and quadratic contrast statements were used to evaluate increasing SID Lys. Differences were considered significant at *P* ≤ 0.05 and a tendency at 0.05 < *P* ≤ 0.10.

## RESULTS

### Sow Gestation Performance

There was an SID Lys × parity group interaction (*P *= 0.039) observed for sow loin depth on day 110 of gestation; however, means did not separate based on the Tukey analysis (Table 2). Increasing SID Lys increased (linear, *P* < 0.001) BW gain from day 90 to 110 of gestation (Table 3). Late gestation SID Lys tended (quadratic, *P* = 0.080) to influence backfat thickness on day 110 of gestation where sows fed 15.8 g/d of SID Lys had a numeric increase in backfat depth compared to sows fed either 11.9 or 19.9 g/d of SID Lys from day 90 to 110 of gestation. Sow loin depth gain over late gestation tended (quadratic, *P* = 0.081) to be smaller for sows fed 15.8 g/d of SID Lys from day 90 to 110 of gestation when compared to sows fed 11.9 or 19.9 g/d of SID Lys. As expected, increasing SID Lys increased (linear, *P* < 0.001) Lys intake/d during late gestation.

**Table 2.** Interactive effects of increased standardized ileal digestible (SID) Lys through increased soybean meal in late gestation and parity on sow lactation and litter performance^1^

Parity group:	Primiparous^2^			Multiparous				*P* =		
SID Lys, g/d^3^:	11.9	15.8	19.9	11.9	15.8	19.9	SEM	Treatment × parity	Treatment	Parity
No. of sows	12	11	12	17	18	17	---	---	---	---
Sow BW change (day 90 to weaning), kg	-7.2	-5.5	4.0	-9.0	-6.6	-10.5	4.67	0.059	0.308	0.025
Sow BW loss (day 110 to weaning), kg	-18.8	-22.2	-13.8	-20.3	-21.1	-27.0	5.32	0.070	0.832	0.096
Sow backfat (weaning), mm	11.7	11.3	11.9	11.7	13.2	12.3	0.75	0.090	0.502	0.047
Sow caliper score (weaning)	12.6^ab^	11.4^b^	12.7^ab^	12.7^ab^	14.1^a^	12.8^ab^	1.11	0.018	0.967	0.022
Sow loin depth (day 110), mm	51.9	49.3	51.0	49.4	51.2	51.5	1.98	0.039	0.547	0.956
Sow urinary creatinine change (day 90 to 110), mg/dL	175.8	-80.6	-224.6	-48.0	79.9	-69.9	114.81	0.087	0.093	0.720
Litter gain (day 10 to weaning), kg	29.3^ab^	28.9^ab^	24.3^b^	27.9^ab^	30.6^a^	29.8^a^	2.38	0.045	0.145	0.080
Litter ADG (day 10 to weaning), kg	3.66^ab^	3.61^ab^	3.03^b^	3.49^ab^	3.82^a^	3.73^a^	0.297	0.045	0.145	0.080
Day 2 to weaning mortality, %	1.3	2.7	4.3	7.7	6.4	4.3	1.90	0.049	0.146	0.015

^a,b^Means within row with different superscripts differ (*P* < 0.05).

^1^A total of 87 mixed-parity sows (Line 241, DNA, Columbus NE) and litters were used from day 90 of gestation until weaning. Litters were cross-fostered within treatment group to equalize litter size within 48-h after farrowing.

^2^Parity group was included as a fixed effect in the data analysis model. Sows were either classified as primiparous (n = 35) or multiparous (n = 52).

^3^Sow treatment consisted of providing a low Lys control diet (SID Lys = 0.60%), a high Lys diet, accomplished by adding additional SBM and L-Lys-HCl (SID Lys = 1.00%), or a 50:50 blend of the two diets (SID Lys = 0.80%) from day 90 of gestation until farrowing. After farrowing, sows were fed a common lactation diet until weaning.

**Table 3.** Effects of increased standardized ileal digestible (SID) Lys through increased soybean meal in late gestation and parity on sow lactation performance^1^

	SID Lys, g/d^2^				SID Lys, *P* =		Parity group^3^			
	11.9	15.8	19.9	SEM	Linear	Quadratic	Primiparous	Multiparous	SEM	Parity, *P* =
No. of sows	29	29	29	---	---	---	35	52	---	---
Parity	1.7	1.7	1.7	0.28	0.970	0.983	1.0	2.9	0.23	0.001
Gestation length, d	117.0	116.9	117.4	0.27	0.196	0.314	117.2	117.0	0.26	0.336
Lactation length, d	18.0	17.9	17.5	0.26	0.106	0.533	17.7	17.8	0.24	0.784
Sow BW, kg										
Day 90	223.5	221.2	221.8	5.18	0.740	0.735	191.8	252.6	5.18	< 0.001
Day 110	235.0	236.6	239.0	4.86	0.428	0.937	207.1	266.6	4.61	< 0.001
Weaning	215.1	215.6	218.5	4.09	0.528	0.794	188.4	244.3	3.69	< 0.001
Gestation gain (day 90 to 110)	11.6	15.4	17.1	1.88	0.001	0.419	15.4	14.0	1.82	0.299
Lactation loss (day 110 to weaning)^4^	−19.6	−21.6	−20.4	4.55	0.798	0.568	−18.3	−22.8	4.42	0.096
Overall loss (day 90 to weaning)^4^	−8.1	−6.0	−3.3	3.86	0.128	0.901	−2.9	−8.7	3.72	0.025
Sow backfat, mm										
Day 90	12.7	13.7	13.5	0.42	0.172	0.222	12.6	14.0	0.37	0.005
Day 110	12.8	13.8	13.2	0.38	0.433	0.080	12.8	13.8	0.34	0.033
Weaning^4^	11.7	12.3	12.1	0.64	0.389	0.419	11.6	12.4	0.62	0.047
Gestation change (day 90 to 110)	0.2	0.1	−0.2	0.36	0.352	0.624	0.3	−0.2	0.34	0.154
Lactation loss (day 110 to weaning)	−1.2	−1.7	−1.1	0.75	0.667	0.043	−1.3	−1.4	0.74	0.458
Overall loss (day 90 to weaning)	−1.0	−1.5	−1.3	0.70	0.487	0.429	−0.9	−1.6	0.68	0.068
Sow caliper score, units										
Day 90	16.2	16.5	16.2	0.57	0.877	0.428	16.1	16.5	0.55	0.139
Day 110	14.9	15.4	15.2	1.16	0.461	0.331	14.9	15.5	1.15	0.118
Weaning^5^	12.6	12.7	12.8	1.02	0.808	0.932	12.2	13.2	1.01	0.022
Gestation loss (day 90 to 110)	−1.2	−1.0	−1.0	0.64	0.367	0.661	−1.1	−1.0	0.63	0.654
Lactation loss (day 110 to weaning)	−2.4	−2.8	−2.5	1.56	0.849	0.254	−2.8	−2.4	1.56	0.145
Overall loss (day 90 to weaning)	−3.6	−3.7	−3.5	1.10	0.770	0.600	−3.9	−3.3	1.09	0.151
Sow loin depth, mm										
Day 90	49.5	50.1	49.6	1.66	0.878	0.543	49.5	50.0	1.63	0.607
Day 110^5^	50.7	50.3	51.2	1.83	0.505	0.380	50.7	50.7	1.81	0.956
Weaning	50.4	50.8	50.9	1.29	0.554	0.815	50.8	50.6	1.26	0.753
Gestation gain (day 90 to 110)	1.2	0.2	1.6	0.62	0.570	0.081	1.2	0.7	0.57	0.465
Lactation change (day 110 to weaning)	−0.4	0.5	−0.4	0.68	0.974	0.112	−0.1	−0.1	0.65	0.986
Overall gain (day 90 to weaning)	0.8	0.7	1.2	0.80	0.683	0.669	1.1	0.7	0.73	0.561
Sow urinary creatinine change (day 90 to 110), mg/dL^4^	63.9	−0.4	−147.3	75.72	0.034	0.627	−43.2	−12.7	69.16	0.720
Sow ADFI, kg										
Gestation (day 90 to farrowing)	1.82	1.84	1.88	0.041	0.150	0.927	1.79	1.90	0.039	0.001
Farrow to weaning	6.40	6.59	6.12	0.253	0.294	0.148	5.69	7.04	0.233	< 0.001
Late gestation average Lys intake, g/d	11.94	15.76	19.88	0.098	< 0.001	0.104	15.71	16.00	0.092	0.001
Wean to estrus interval, d	5.4	5.4	5.2	0.09	0.205	0.412	5.3	5.3	0.08	0.551

^1^A total of 87 mixed-parity sows (Line 241, DNA, Columbus, NE) and litters were used from day 90 of gestation until weaning. Litters were cross-fostered within treatment group to equalize litter size within 48-h after farrowing. No treatment × parity group interactions (*P* > 0.10) were observed unless otherwise noted.

^2^Sow treatment consisted of providing a low Lys control diet (SID Lys = 0.60%), a high Lys diet, accomplished by adding additional SBM and L-Lys-HCl (SID Lys = 1.00%), or a 50:50 blend of the two diets (SID Lys = 0.80%) from day 90 of gestation until farrowing. After farrowing, sows were fed a common lactation diet until weaning.

^3^Parity group was included as a fixed effect in the data analysis model. Sows were either classified as primiparous (n = 35) or multiparous (n = 52).

^4^Treatment × parity group, *P* < 0.10.

^5^Treatment × parity group, *P* < 0.05.

Multiparous sows were heavier and had greater backfat at both day 90 and 110 (*P* ≤ 0.033) and consumed more feed during gestation (*P* = 0.001), resulting in slightly higher SID Lys intake (*P* = 0.001) compared to primiparous sows.

### Sow Lactation Performance

There was a tendency for a treatment × parity group interaction (*P *≤ 0.070) observed for sow BW loss from day 110 of gestation until weaning and sow BW loss from day 90 of gestation until weaning (Table 2). Primiparous sows fed 19.9 g/d SID Lys gained BW over lactation, while those fed lower levels lost BW. A treatment × parity group interaction (*P* = 0.018) was observed for caliper score at weaning, where primiparous sows fed 15.8 g/d had lower caliper scores than multiparous sows fed the same level with all others intermediate. A similar tendency for a treatment × parity group interaction (*P* = 0.090) was observed for backfat depth at weaning (P = 0.090), though means did not separate. During lactation, sows fed 15.8 g/d of SID Lys during late gestation had an increase (quadratic, *P* = 0.043) in backfat loss compared to those fed 11.9 or 19.9 g/d of SID Lys (Table 3). Wean to estrus interval (WEI) was unaffected by SID Lys intake from day 90 of gestation until farrowing.

Multiparous sows lost (*P* = 0.025) more weight from day 90 of gestation until weaning and tended (*P* ≤ 0.096) to lose more weight during lactation and backfat from day 90 of gestation until weaning compared to primiparous sows. However, multiparous sows remained heavier (*P* < 0.001) than primiparous sows at weaning. Multiparous sows had greater lactational ADFI when compared to primiparous sows (*P* < 0.001).

### Litter Performance

There was a treatment × parity group interaction (*P* = 0.045) observed for litter gain and litter ADG from day 10 to weaning (Table 2). Primiparous sows previously fed 19.9 g/d of SID Lys from day 90 of gestation until farrowing had the lowest litter gain and litter ADG, while multiparous sows fed either 15.8 or 19.9 g/d of SID Lys had the greatest litter gain. There was a treatment × parity group interaction (*P* = 0.049) on day 2 to weaning mortality which increased with higher SID Lys in primiparous sows but decreased with higher SID Lys in multiparous sows.

The percentage of pigs born alive tended (linear, *P* = 0.093) to decrease and stillborns tended (linear, *P* = 0.068) to increase as SID Lys intake in late gestation increased (Table 4). When litter birth weight was divided by litter size, there were no effects of SID Lys level from day 90 of gestation until farrowing on pig birth weight. Litter ADG from day 2 to 10 tended (linear, *P* = 0.060) to increase, as did piglet ADG (linear, *P* = 0.014), as SID Lys fed from day 90 of gestation until farrowing increased, but there were no differences from day 10 to weaning or overall. Sows fed 15.8 g/d of SID Lys from day 90 of gestation until farrowing had the greatest (quadratic, *P* = 0.044) litter gain from day 2 to weaning and tended (quadratic, *P* = 0.075) to have the heaviest litter weaning weights when compared to litters from sows fed 11.9 or 19.9 g/d of SID Lys.

**Table 4.** Effects of increased standardized ileal digestible (SID) Lys through increased soybean meal in late gestation and parity on litter performance^1^

	SID Lys, g/d^2^				SID Lys, *P* =		Parity group ^3^			
	11.9	15.8	19.9	SEM	Linear	Quadratic	Primiparous	Multiparous	SEM	Parity, *P* =
Litter characteristics										
Total born, n	16.0	16.3	15.7	0.78	0.754	0.630	14.0	18.3	0.64	0.010
Born alive, %	91.3	92.3	87.3	1.79	0.093	0.155	94.1	85.2	1.12	0.005
Stillborn, %	5.0	4.9	8.4	1.40	0.068	0.301	4.4	8.0	0.96	0.115
Mummy, %	3.5	2.6	3.5	1.25	0.995	0.512	1.4	6.9	0.82	0.025
Litter size, n										
Day 1	14.6	15.0	14.0	0.75	0.548	0.465	14.0	15.1	0.64	0.511
Day 2	14.2	14.1	13.8	0.72	0.651	0.894	13.6	14.5	0.63	0.568
Day 10	13.6	13.8	13.3	0.71	0.723	0.732	13.3	13.8	0.63	0.874
Weaning	13.6	13.6	13.2	0.72	0.706	0.804	13.3	13.7	0.63	0.759
Litter weight, kg										
Day 1	23.0	21.9	20.9	0.87	0.080	0.974	19.3	24.6	0.79	< 0.001
Day 2	23.6	23.2	22.3	0.80	0.117	0.718	21.3	24.8	0.76	< 0.001
Day 10	43.1	45.1	42.9	1.47	0.909	0.183	41.7	45.7	1.35	0.008
Weaning	72.0	75.0	69.8	1.96	0.419	0.075	69.0	75.5	1.77	0.003
Mean pig BW, kg										
Day 1	1.57	1.47	1.50	0.068	0.438	0.430	1.38	1.65	0.061	0.001
Day 2	1.66	1.65	1.63	0.044	0.632	0.945	1.58	1.71	0.039	0.007
Day 10	3.16	3.30	3.25	0.141	0.443	0.356	3.15	3.32	0.136	0.089
Weaning	5.29	5.53	5.34	0.154	0.786	0.193	5.22	5.55	0.141	0.046
Litter gain, kg										
Day 2 to day 10	19.5	21.9	20.6	1.37	0.468	0.152	20.4	21.0	1.28	0.656
Day 10 to weaning^4^	28.6	29.7	27.0	2.76	0.254	0.112	27.5	29.4	2.04	0.080
Day 2 to weaning	48.2	51.9	47.5	1.62	0.768	0.044	47.7	50.7	1.44	0.110
Litter ADG, kg										
Day 2 to day 10	2.45	2.75	2.79	0.130	0.060	0.398	2.64	2.68	0.117	0.765
Day 10 to weaning^4^	3.58	3.71	3.38	0.261	0.254	0.112	3.43	3.68	0.255	0.080
Day 2 to weaning	2.69	2.90	2.73	0.109	0.748	0.075	2.69	2.86	0.101	0.100
Pig ADG, g										
Day 2 to day 10	180	202	213	13.4	0.014	0.617	202	194	12.7	0.481
Day 10 to weaning	262	273	259	16.6	0.824	0.263	260	270	16.0	0.335
Day 2 to weaning	197	214	210	8.0	0.219	0.201	204	210	7.4	0.512
Within litter BW CV, %										
Day 1	18.2	17.6	18.6	1.47	0.805	0.557	16.5	19.8	1.38	0.011
Day 2	18.4	19.2	20.9	1.42	0.089	0.734	18.4	20.6	1.34	0.089
Day 10	17.7	19.5	16.8	0.93	0.498	0.044	17.0	19.0	0.83	0.060
Weaning	17.5	18.2	16.2	1.03	0.380	0.278	16.6	18.0	0.91	0.260
Milk yield^5^										
Total yield (day 2-weaning), kg	199.8	204.5	189.4	6.75	0.224	0.186	192.6	203.2	6.15	0.132
Average yield, kg/d	12.5	12.9	12.2	0.51	0.528	0.213	12.2	12.9	0.49	0.057
Colostrum protein content, %	18.9	19.4	19.3	0.43	0.545	0.590	19.2	19.1	0.39	0.828
Preweaning mortality, %										
Birth to day 2	1.9	4.6	2.0	1.35	0.959	0.018	1.8	3.7	0.97	0.150
day 2 to weaning^4^	3.2	4.2	4.8	1.17	0.339	0.879	2.7	6.0	0.88	0.015

^1^A total of 87 mixed-parity sows (Line 241, DNA, Columbus NE) and litters were used from day 90 of gestation until weaning. Litters were cross-fostered within treatment group to equalize litter size within 48-h post farrowing. No treatment × parity group interactions (*P* > 0.10) were observed unless otherwise noted.

^2^Sow treatment consisted of providing a low Lys control diet (SID Lys = 0.60%), a high Lys diet, accomplished by adding additional SBM and L-Lys-HCl (SID Lys = 1.00%), or a 50:50 blend of the two diets (SID Lys = 0.80%) from day 90 of gestation until farrowing. After farrowing, sows were fed a common lactation diet until weaning.

^3^Parity group was included as a fixed effect in the data analysis model. Sows were either classified as primiparous (n = 35) or multiparous (n = 52).

^4^Treatment × parity group, *P* < 0.05.

^5^Total milk yield was estimated utilizing the model developed by Hansen et al. (2012). Average milk yield = Total milk yield / (Wean age—2).

Litter CV for pig BW did not differ on day 1 of lactation (Table 4). However, litter CV for pig BW tended (linear, *P* = 0.089) to increase on day 2 with increasing SID Lys fed in late gestation and was greater on day 10 in litters from sows fed 15.8 g/d SID Lys (quadratic, *P* = 0.044), with no differences at weaning. Litters from sows fed 15.8 g/d of SID Lys had the greatest (quadratic, *P* = 0.018) birth to day 2 piglet mortality compared to sows fed either 11.9 or 19.9 g/d of SID Lys from day 90 of gestation until farrowing. Calculated total and average daily milk yield did not differ based on SID Lys level fed in late gestation or by parity group.

The total number of pigs born was greater, percentage of pigs born alive was lower, and incidence of mummies was greater (*P* ≤ 0.025) in multiparous sows compared to primiparous sows (Table 4). Individual piglet BW and litter weight in multiparous sows was greater (*P* < 0.05) at all timepoints. Litter ADG from day 10 to weaning and day 2 to weaning tended (*P* ≤ 0.100) to be greater in multiparous sows than in primiparous sows, but litters from primiparous sows tended (P ≤ 0.089) to have lower litter CV for pig BW on day 2 and day 10. Multiparous sows tended (*P* = 0.057) to have greater average daily milk yield than primiparous sows.

### Colostrum and Urine Analyses

Colostrum protein content did not differ based on late gestation diet (Table 4). There was a tendency for a treatment × parity group interaction (*P* = 0.087) observed for change in urinary creatinine from day 90 to day 110 of gestation (Table 2). The change in urinary creatinine concentration progressed from positive to negative as SID Lys in late gestation increased in primiparous sows, whereas in multiparous sows, the SID Lys level in late gestation diets did not influence the change in urinary creatinine. Overall, increasing SID Lys intake in from day 90 until day 110 of gestation reduced (linear, *P* = 0.034) the magnitude of the decrease in urinary creatinine concentration over time (Table 3).

## DISCUSSION

This trial increased SID Lys in sow diets fed from day 90 of gestation until farrowing through additional SBM. While SBM also contributes crude protein and other AA, based on AA:Lys ratios, Lys remained the first limiting AA.

Farmer et al. (2022) evaluated the effects of a 40% increase in SID Lys above NRC (2012) requirement estimates through increasing SBM in diets for primiparous sows. They observed that fetal weight, total mammary parenchymal mass, and total parenchymal fat, protein, DNA, and RNA increased as SID Lys intake increased from 18.6 to 26.0 g/d. However, in a second study, Farmer et al. (2023) observed no differences in fetal weight or mammary development in multiparous sows with the same 40% increase in SID Lys. In both studies, sows were euthanized at day 110 of gestation, preventing evaluation of litter performance. Kloostra et al. (2025) further increased SID Lys via SBM (70 to 160% of NRC (2012) requirement estimates) in primiparous sows and observed that milk yield peaked at 120% of NRC (2012) requirement estimates, while whole-body N retention and piglet birth weight were optimized at 115%.

The SID Lys requirement for late gestating sows is not well established, with previous studies reporting differing estimates based on the specific indicators evaluated. Jo and Kim (2023) estimated the late gestation (day 77 to 103) SID Lys requirement of multiparous sows (parity = 5.1 ± 2.0) to be 16.1 g/d based on total pigs born alive. Conversely, Thomas et al. (2021) using whole-body protein deposition modeling, suggested 11.0 g/d throughout gestation for both primiparous and multiparous sows, with the exception of the last 5- to 10-d of gestation when sows enter a negative SID Lys-balance and need to be provided with at least 13.5 g/d of SID Lys. Samuel et al. (2012) used the indicator of AA oxidation method with simultaneous determination of heat production and reported late gestating requirements (day 86 to 110 of gestation) of 18.7 g/d and 13.0 g/d for second- and third-parity sows, respectively, and recommended parity segregation to improve precision.

As reported in previous studies (Heo et al., 2008; Yang et al., 2009; Gonçalves et al., 2016; Che et al., 2019) and in agreement with the results of the present study, increased SID Lys intake supported greater BW gain in late gestation. Similar to this trial, Farmer et al. (2022) and Kloostra et al. (2025), increased SID Lys through additional SBM and also observed greater late gestation (day 90 to 110 of gestation) BW gain in primiparous sows but no differences were observed in multiparous sows (Farmer et al., 2023).

The effects of increased SID Lys in late gestation on backfat change are more variable. Heo et al. (2008) and Yang et al. (2009) observed an increase in backfat gain when SID Lys increased; while others, similar to the current trial, saw no difference based on late gestation diet (Farmer et al., 2022, 2023). Variability in this response criteria may linked to method of SID Lys supplementation where studies that achieved higher SID Lys through L-Lys-HCl reported an increase in backfat, whereas in studies that used SBM there was no effect. Differences in digestion of Lys when it is supplemented as a free AA compared with protein-bound Lys could be involved. Free AA are absorbed more rapidly than protein-bound AA (Batterham, 1985). This could result in differences in Lys utilization, especially when gestating sows are only fed once daily, as protein synthesis requires that all necessary AA are available at the same time. Thus, the additional Lys supplied by SBM in the diet may have been utilized for protein synthesis in fetal or maternal tissues whereas the additional Lys supplied by L-Lys-HCl may not have been utilized for protein synthesis and may have instead been converted to glucose through gluconeogenesis.

Despite increased parenchymal mass, which led to increased total protein, total DNA, and total RNA reported by Farmer et al. (2022), we did not observe increased colostrum protein content in response to higher SBM in late gestation diets, consistent with Yang et al. (2009), Che et al. (2019), and Johannsen et al. (2024). Conversely, both Yang et al. (2008) and Heo et al. (2008) observed increased colostrum protein content when sows were fed high levels of SID Lys in late gestation.

Late gestating sows may have to mobilize and catabolize their own muscle protein to meet the demands of fetal and mammary growth during late gestation. Creatinine is generated as a metabolic waste product during the process of muscle catabolism and therefore serves as a marker of protein mobilization. The decrease in urinary creatinine in the present study as SID Lys increased may point to lower muscle catabolism. Heo et al. (2008) measured blood creatinine levels post-farrowing and observed a decrease in creatinine levels in primiparous sows when SID Lys intake was increased from 18 g/d to 24 g/d beginning on day 80 of gestation. This is contrary to results by Yang et al. (2008, 2009) who observed no differences in blood creatinine levels post-farrowing with increased SID Lys in late gestation diets.

In contrast to the present study, Yang et al. (2009) observed an increase in pig birth weight, while Kloostra et al. (2025) reported an increase in pig birth weight when SID Lys was increased through additional SBM up to 22 g/d, after which pig birth weight decreased once again. The increase in stillbirths observed in the present study with increasing SID Lys intake from day 90 of gestation until farrowing is consistent with the findings of Johannsen et al. (2024), who reported a linear increase in stillbirths when SID Lys intake was elevated from 14.3 g/d to 31.4 g/d during the transition period (day 108 until farrowing). However, dissimilar to the current study, Johannsen et al. (2024) also reported a linear increase in total born pigs as SID Lys intake increased which likely contributed to the increase in stillbirths. This outcome contrasts with other studies that reported either no effect on stillbirths (Che et al. 2019) or a reduction in stillbirths (Gonçalves et al., 2016; Thomas et al., 2021b) when SID Lys intake in late gestation was increased through added SBM. It is worth noting that previous research has also linked high crude protein intake in late gestation to increased stillbirth rates and prolonged farrowing duration, potentially due to the greater heat increment associated with metabolizing additional protein (Tydlitát et al., 2008).

Similar to results of Gonçalves et al. (2016), there were no differences in birth weight litter CV in the current study as SID Lys intake increased. It is unclear as to why differences in litter CV for pig BW were observed at only day 2 and 10 but no effect was observed at day 1 or weaning. However, the cross-fostering technique used in the current study may have impacted the litter CV for pig BW early in lactation.

Given that increased SID Lys intake in late gestation improved mammary development in primiparous sows but not in multiparous sows (Farmer et al., 2022, 2023), we hypothesized a potential interaction between SID Lys level and parity group on milk yield and offspring growth. Indeed, litter ADG from day 10 to weaning was influenced by a parity group × SID Lys interaction, though the reason for reduced litter ADG in primiparous sows fed 19.9 g/d SID Lys remains unclear, particularly given that increasing SID Lys intake in multiparous sows led to improved litter ADG over the same period. Che et al. (2019) observed an improvement in overall pig ADG when sows were fed 20.6 g/d of SID Lys compared to 14.7 g/d of SID Lys in late gestation while in the present study litter ADG was optimized in sows fed 15.8 g/d of SID Lys in late gestation. Surprisingly, although there were differences in litter and pig ADG at various time points due to gestation dietary treatment, there were no differences in calculated total milk yield or average milk yield. This may be due, in part, to numerical differences in litter size between treatments. In contrast, Johannsen et al. (2024) observed that in multiparous sows, milk yield increased when SID Lys intake increased (through additional SBM) from 14.3 g/d until the breakpoint of 21.4 g/d in transition diets fed from day 108 of gestation until farrowing. Similarly, Kloostra et al. (2025) found that in primiparous sows fed increasing levels of SID Lys through additional SBM from day 90 of gestation until farrowing, milk yield was maximized at 23.0 g/d.

The quadratic increase in PWM within the first 48 hours in the current study may be related to the numerical increase in the number born alive observed in sows fed 15.8 g/d of SID Lys. Larger litters typically reduce colostrum intake per pig, increasing the risk of crushing or starvation (Edwards and Baxter, 2015). The cause of the interaction on day 2 to weaning mortality, is unclear and warrants further investigation with a larger sample size. The present study contrasts with previous studies which have reported either no effect (Che et al., 2019) or a reduction in PWM (Gonçalves et al., 2016) when SID Lys was increased through increased SBM.

Consistent with past literature (Che et al., 2019), increasing SID Lys in late gestation did not affect lactation backfat depth loss, lactation ADFI, or WEI. In the current trial, the interaction between SID Lys intake in late gestation and parity group on sow BW change from day 90 of gestation until weaning could be due, in part, to differences in average litter size at weaning. In addition, primiparous sows in this trial were still growing, and thus, additional SID Lys was more beneficial for these sows as opposed to multiparous sows.

In conclusion, increasing SID Lys intake from 11.9 to 19.9 g/d from day 90 of gestation to farrowing increased late gestation BW gain and early lactation pig ADG. Overall litter gain and litter weaning weight were optimized when sows were fed 15.8 g/d of SID Lys from day 90 of gestation until farrowing. Results from this study suggest that the NRC (2012) requirement estimates for primiparous sows (16.7 g/d) appears to be adequate to optimize lactation performance.
